# RDA-Genebank and Digital Phenotyping for Next-Generation Research on Plant Genetic Resources

**DOI:** 10.3390/plants12152825

**Published:** 2023-07-31

**Authors:** Seong-Hoon Kim, Parthiban Subramanian, Young-Wang Na, Bum-Soo Hahn, Yoonha Kim

**Affiliations:** 1 National Agrobiodiversity Center, National Institute of Agricultural Sciences, RDA, Jeonju 5487, Republic of Koreaywna@korea.kr (Y.-W.N.); bshahn@korea.kr (B.-S.H.); 2 Laboratory of Crop Production, Department of Applied Biosciences, Kyungpook National University, Daegu 41566, Republic of Korea

**Keywords:** genebank, digital phenotyping, Nagoya Protocol, agronomic traits

## Abstract

The National Agrobiodiversity Center under the Rural Development Administration (RDA) in Jeonju, Republic of Korea stands as the foremost national genebank in the country. Over the years, the National Agrobiodiversity Center has remained committed to enriching its collection with foreign genetic resources, elevating its status to a world-class plant genetic resources (PGR)- holding genebank. Currently, several steps are being undertaken to improve the accessibility of the collection to national as well as international researchers, improve the data available on the resources, and amend the passport information for the accessions. With the implementation of the Nagoya Protocol, the origin of genetic resources is being highlighted as an important input in the passport information. The RDA-Genebank actively responds to the Nagoya Protocol by supplementing passport data for resources lacking information on their origin. In addition, a large number of conserved resources are continuously multiplied, and agronomic traits are investigated concurrently. With the traditional methods of characterization of the germplasm requiring a significant amount of time and effort, we have initiated high-throughput phenotyping using digital techniques to improve our germplasm data. Primarily, we have started adding seed phenotype information followed by measuring root phenotypes which are stored under agronomic traits. This may be the initial step toward using largescale high-throughput techniques for a germplasm. In this study, we aim to provide an introduction to the RDA-Genebank, to adopted international standards, and to the establishment of high-throughput phenotyping techniques for the improvement of passport information.

## 1. Introduction to the National Agrobiodiversity Center (RDA-Genebank)

**Figure 1:**
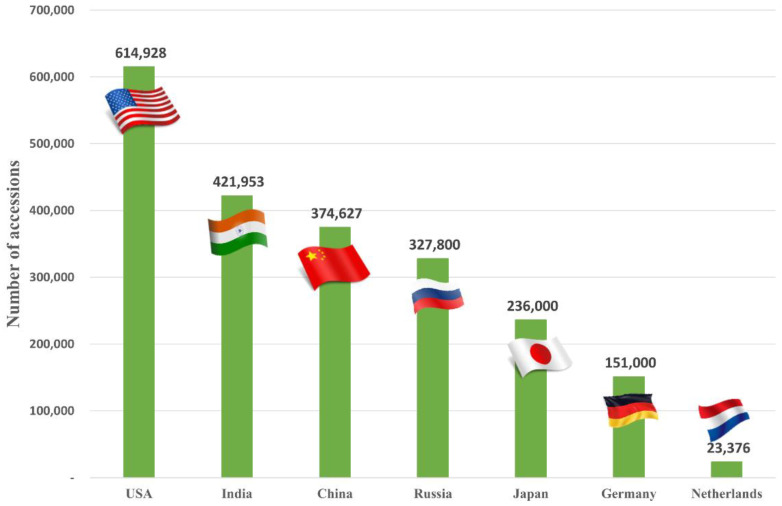
Countrywide statistics of the number of germplasms conserved at their international genebanks across the world [1,2,3,4,5,6].

The National Agrobiodiversity Center in Jeonju, Republic of Korea, under the administration of the Rural Development Administration (RDA), plays a pivotal role in the collection, evaluation, conservation, and distribution of diverse plant genetic resources, including both seeds and vegetative germplasms. With the establishment of a low-temperature seed storage facility in 1975 and a seed management office in 1976, around 3300 germplasms were managed at local agricultural institutions. Later, in 1980, official documentation of 12,865 rice germplasms conserved by local agricultural institutions and universities was carried out, followed by their addition to the low-temperature seed storage collection. While seed germplasms were conserved at the genebank, external management institutions managed vegetative germplasms. In 1993, the RDA-Genebank began international cooperation starting with the N.I. Vavilov All-Russian Institute of Plant Genetic Resources (VIR) (1993–2019). At the RDA-Genebank, researchers aim to secure germplasm diversity by continuously collaborating with international joint research institutes including the Uzbek Research Institute of Plant Industry (UzRIPI) in Uzbekistan (1996–2004), the Department of Agriculture (DOA) Genebank in Thailand (1998), the Leibniz Institute of Plant Genetics and Crop Plant Research (IPK) (1998–1999) in Germany, the International Rice Research Institute (IRRI) in the Philippines, the Plant Science and Agricultural Research Institute (PASRI) in Mongolia, the National Bureau of Plant Genetic Resources (NBPGR) in India, and the Georgian Academy of Agricultural Sciences (GAAS) in Georgia. Adoption of international policies such as the Convention on Biological Diversity (CBD) at the United Nations conference on environment and development (UNCED) in 1992 and the Nagoya Protocol on Access to Genetic Resources and the Fair and Equitable Sharing of Benefits Arising from Their Utilization in 2010 has further reinforced germplasm conservation efforts. Despite the limitations on data disclosure and international distribution of germplasms, Korea remains one of the significant countries conserving plant biodiversity. Figure 1 presents a count of conserved germplasms in international genebanks. This data was gathered from Genesys and the respective homepages of each country. In adherence to Korea’s policy at this time, the RDA-Genebank is unable to disclose the specific quantity of germplasms under conservation. However, more than 2.7 million accessions had been conserved as of June 2023.

## 2. RDA-Genebank and Nagoya Protocol

The Nagoya Protocol is an international agreement that aims to promote the fair and equitable sharing of benefits arising from the use of genetic resources, including those held in genebanks. The protocol was adopted in 2010 under the United Nations Convention on Biological Diversity (CBD) and entered into force in 2014 [7]. The history of the Nagoya Protocol can be traced back to the CBD, which was adopted at the 1992 Earth Summit in Rio de Janeiro, Brazil. The CBD recognized the importance of conserving biodiversity and the need to ensure the fair and equitable sharing of benefits arising from the use of genetic resources [8]. However, it was found that the existing legal framework did not adequately address the issue of benefit sharing. This led to the development of the Nagoya Protocol, which provides a framework for the fair and equitable sharing of benefits arising from the utilization of genetic resources. The protocol establishes rules for access to genetic resources, including prior informed consent and mutually agreed terms, and requires users to share the benefits arising from the use of those resources with the providers. In the context of genebanks, the Nagoya Protocol has important implications for the collection and utilization of genetic resources. Genebanks are required to comply with the access and benefit-sharing requirements of the protocol when obtaining new genetic resources, and must also ensure that any benefits arising from the use of those resources are shared fairly and equitably with the countries and communities that provided them [8].

Overall, the Nagoya Protocol represents a significant milestone in the international effort to promote the conservation and sustainable use of genetic resources, including those held in genebanks, and to ensure that the benefits of these resources are shared fairly and equitably. In accordance with the Nagoya Protocol, RDA-Genebank continuously updates passport data on the origins of germplasms to actively respond to the utilization of genetic resources and the sharing of benefits accordingly. As a method of supplementing origin information, the passport data disclosed on the homepage of the relevant genebank was reflected on the basis of the original international genebank resource number.

The website of our institution presently offers visitors the option to select either the Korean or the English version (http://genebank.rda.go.kr/plantMain.do, accessed on 23 Mar 2023). Whereas the Korean version provides access to essential passport data, including conserved genetic resources of plants for distribution, the English version falls short in divulging the current status of resource conservation and the pertinent passport data. Consequently, this omission poses challenges for foreign researchers seeking to obtain and investigate our germplasms.

## 3. RDA-Genebank’s Distribution of Germplasms

**Figure 2:**
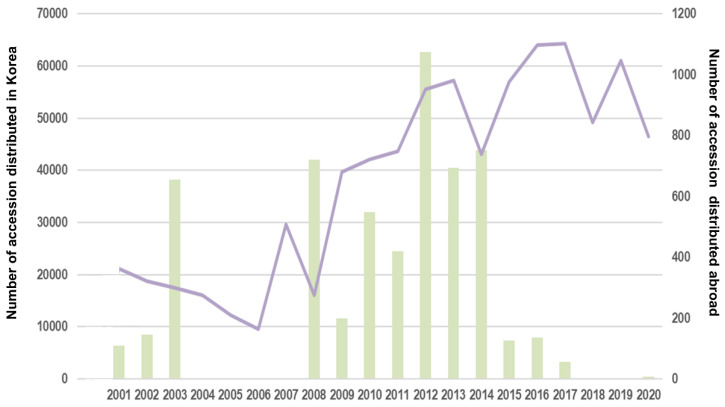
Statistics on germplasm dissemination by RDA-Genebank in the past 20 years from 2001 to 2020. The purple trendline shows germplasm distributed domestically (Korea), and the green columns show germplasm distributed abroad.

Over the past two decades, the RDA-Genebank has distributed an impressive 770,280 accessions at an annual rate of 38,514 accessions on average. Nearly all of these accessions (99.27%) were domestically distributed in the Republic of Korea, while only a small proportion (0.73%) was circulated internationally (Table 1). The trend of domestic distribution has remained persistently upwards, with a minor downward fluctuation in 2008 that may be attributed to the global economic crisis. However, the subsequent year witnessed a substantial rise in distribution, potentially attributable to the base effect (Figure 2).

In 2018, the RDA-Genebank initiated a collaboration research program, the Technology Development Project for Customized Agricultural, Forestry, and Fishery Products and Services, partnering with agricultural organizations such as seed companies and private research institutions. The objectives of this program were to broaden and improve the characterization and evaluation information of the conserved germplasms, augment their diversity, propagate them, and concurrently conduct characterization research. Through chemical composition profiling or disease resistance evaluation, commendable genetic resources have been shortlisted from the outcomes of these research endeavors, to be recommended for breeding and further scientific investigations. During 2010–2013, a total of 1343 accessions were distributed for local propagation of rice to the IRRI, the Philippines. For the purpose of germplasm exchange with international genebanks, the RDA-Genebank has introduced 568 accessions in the ARS (2012) in the US, 995 accessions in the UzRIPI in Uzbekistan (2011–2014), 10 in the VIR in Russia (2014), 235 in the AVRDC in Taiwan (2013–2016), and 232 in the GAAS in Georgia (2015–2016) as a part of its germplasm exchange policy. We have also supplied to private entities, including the distribution of 62 sesame accessions to the Tainan District Agricultural Research and Extension Station in Taiwan. The distribution overseas can be categorized into two types: those sent through Korea Project to the International Agriculture (KOPIA) center as part of Official Development Assistance (ODA) projects, which provide country-specific farming techniques, and those distributed through the 3-FACI. The 3-FACI initiative includes the Asian Food & Agriculture Cooperation initiative (AFACI), the Korea–Africa Food & Agriculture Cooperation initiative (KAFACI), and the Korea–Latin America Food & Agriculture Cooperation initiative (KoLFACI). These programs aim to facilitate multi-continental research and collaboration to address common farming problems. Despite RDA-Genebank’s possession of world-class germplasms, limited overseas distribution has occurred due to the unavailability of individual resource information on online platforms such as Genesys, managed by the Global Crop Diversity Trust. Exceptions to this pattern have occurred only when researchers with Korean affiliations have utilized publicly available history information to apply for resource distribution. Our institution is diligently making preparations, including the dissemination of passport information, to ensure the continuous availability and accessibility of our plant genetic resources for international distribution.

## 4. Passport Data and Agronomic Traits

**Figure 3:**
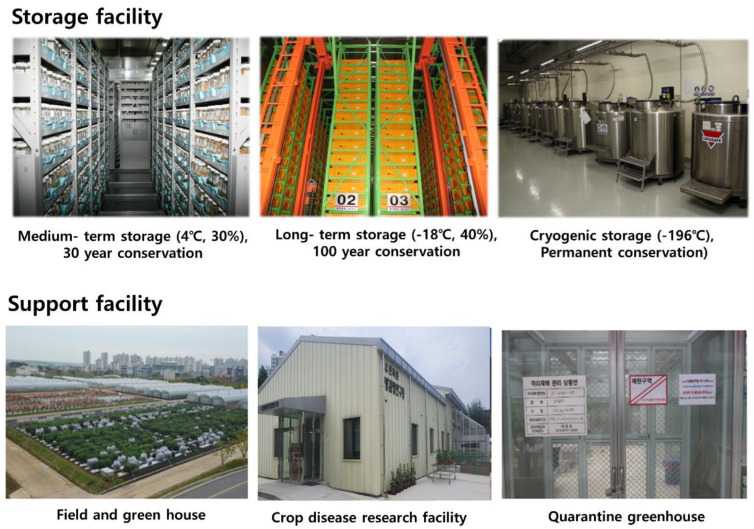
Storage facilities and support facilities installed by RDA-Genebank for germplasm management (temperatures are given as degree Celcius, and humidity maintained is given as % relative humidity).

In order to improve the utilization of the conserved germplasms, systematic passport data management is indispensable [9]. Germplasms deposited in the RDA-Genebank are stored in cold storage after verification of the seed viability (Figure 3). Users of the genebank, including breeders and researchers, are provided with technical support to select germplasms on the basis of passport data so that they may find the appropriate germplasm for their purpose. Germplasms deposited through domestic and overseas field collections and institutional introductions are managed by initially assigning temporary numbers in the RDA-Genebank. For domestic and overseas field-collected germplasms, the passport data prepared by the collector are adopted, and for the introduction of germplasms held by domestic and foreign institutions, a database is prepared by reflecting the passport data held by the existing institutions. In the period 1985–2004, a database was established including 14 descriptors, including crop type (food, horticulture and specialty crop, etc.), origin, scientific name, germplasm name, and status (cultivar, landrace, and wild, etc.) for the passport data of 150,000 accessions collected and introduced to the RDA-Genebank. In order to manage temporary germplasms as nationally registered resources, the number of seeds and viability standards established for each crop must be met. Through the periodic propagation project, the viability and seed count are updated, and characterization data for each crop are created according to the standards of the RDA-Genebank.

In response to consumer preferences, there is a notable increase in the incorporation of evaluation data concerning chemical composition profiles, such as glucosinolate levels in Brassica ssp. and disease resistance. This highlights the importance of considering these factors in the evaluation process. Since the 1990s, the RDA-Genebank has systematically investigated its conserved genetic resources for their qualitative and quantitative traits to promote the use of germplasms by consumers of the end product. During the early stages, the characterization centered on seed viability and primary characteristics of germplasms such as seed coat color, width, and length. From 1995, the organization was subdivided into several divisions for cereal crops, horticultural crops, and special crops, respectively, and their evaluation data was augmented. In addition, chemical composition profiles such as the oil and fatty acid content of sesame seeds; the protein content, amylose and amylopectin content, and starch characteristics of rice landraces; and the DNA polymorphism analysis of rice and barley were initiated [10]. In the soybean (*Glycine max*), the morphological characteristics of 650 landraces were investigated, about 40 of them were selected as a core collection, and a molecular genetic profile was promoted [11]. Among the recent data updates of the passport information, significant achievements include the selection of strains resistant to the blight of red pepper (*Capsicum* spp.), the analysis of capsaicin content, the cultivation of anthracnose-resistant intermediate seedlings, the evaluation of Chinese cabbage (*Brassica rapa* ssp. *penkinensis*) landraces, the cultivation of superior groups, and the cultivation of intermediate seedlings resistant to wart disease of cabbage [12]. The germplasms evaluated until the year 2000 accounted for 79% of the total germplasms; rice, barley, miscellaneous grains, and legumes were evaluated at 88–68%, while horticultural crops were less than 54%.

**Figure 4:**
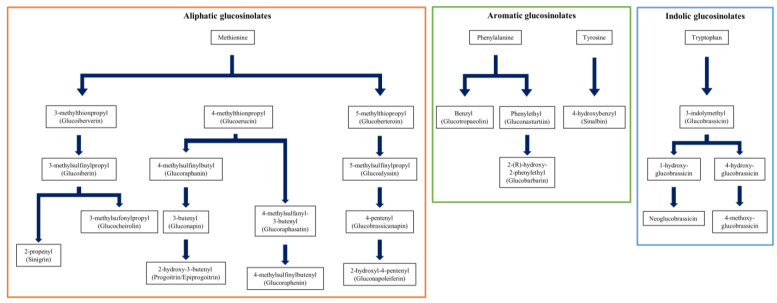
Three major biosynthesis pathways of glucosinolate in *Brassicaceae* [14].

Starting in 2019 with Chinese cabbage (*B. rapa* subsp. *pekinensis*), our studies focused on analyzing the individual glucosinolates of the mustard leaf (*B. juncea*) and the Choy Sum (*B. rapa* subsp. *chinensis var. parachinensis*) [13,14]. The glucosinolate synthetic pathway comprises three distinct pathways, and for the first time we conducted a correlation analysis between these pathways through individual glucosinolate analysis within our genebank (Figure 4). Furthermore, our research revealed that one of the germplasms, IT228140, exhibited the capacity to synthesize significant quantities of glucobrassicanapin and progoitrin. These specific glucosinolates have been associated with various therapeutic applications. The identification of these conserved germplasms as potential bioresources holds immense promise for breeders. By providing information on therapeutically significant glucosinolate content, we contribute to the development of plant varieties that can naturally enhance public health outcomes.

With further work carried out on expanding the information available in the evaluation data for germplasms conserved at the RDA-Genebank, substantial consideration has been given to using high-throughput techniques to study the germplasms. One such aspect is using digital phenotyping for largescale analysis and screening of the germplasms.

## 5. Digital Phenotyping for Next-Generation Research on Plant Genetic Resources

**Figure 5:**
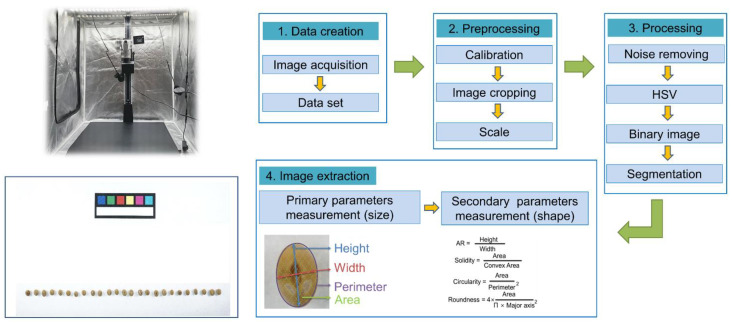
Flow chart of seed phenotyping using 2D image created by digital camera currently used in RDA-Genebank [20].

The significance of seed traits is widely acknowledged, and seeds are selected on the basis of their phenotypic characteristics to improve crop yield and quality, making this one of the oldest phenotyping techniques [15]. Phenotypic characteristics, such as seed size and shape, are considered important in agriculture, as they have a direct influence on consumer preference and market value. Consequently, traditional breeders aim for high yield, specific grain size, and shape, with long grains being preferred for rice, and large, spherical grains being favored for wheat due to their suitability for milling [16]. With the exponential growth of genomic information about plants, advanced phenotyping techniques are now required to complement the genotype data obtained from next-generation sequencing technology [17]. Phenomics technology also has made significant progress in the past ten years in various aspects. However, the process of building phenotypic databases is time-consuming, resulting in a phenotypic bottleneck [18]. By constructing two-dimensional (2D) images using cameras and scanners as well as three-dimensional (3D) images using techniques such as magnetic resonance imaging (MRI) and computed tomography (CT), it has now become possible to analyze crop phenotypes in a detailed manner [19]. However, 3D imaging can be expensive and is not always practical, particularly for the large numbers of genetic materials conserved in genebanks. By contrast, 2D imaging is a rapid and cost-effective method that can be used for a large number of germplasms. In this regard, the RDA-Genebank has adopted the use of ImageJ Software, utilizing 2D images constructed with RGB cameras to phenotype seeds (Figure 5). This method has been proven successful, with a high correlation coefficient (*r*^2^) between image-based and actual height and the width measurements of soybean seeds (*r*^2^ = 0.9735, *r*^2^ = 0.9839, respectively) [20]. Therefore, this technique can be applied to a large number of plant genetic resources conserved in the international genebank. In 2022, eight seed phenotype characteristics were measured using digital analysis techniques in 598 soybeans (*Glycine max*), and diversity analysis was performed using origin (Republic of Korea, China, and the USA) and status (landrace, cultivar, and wild) [20]. Currently, 2D methods are employed to compare seed traits of Northeast Asian landraces, including the Republic of Korea, China, North Korea, and Japan, which are recognized as the primary producers of soybeans. Furthermore, chemical composition profiling, such as antioxidant activity, is incorporated to validate the correlation between seed traits [21]. In genebanks, it is imperative not only to include the agronomic traits sought by consumers but also to anticipate future research directions and continuously incorporate characteristic investigation items. Roots, which are fundamental plant organs responsible for absorbing water and nutrients, are crucial for supporting soil, crop productivity, and environmental stress acclimation such as drought, and as such are indispensable plant organs for breeders. Nonetheless, roots are more challenging to study when compared to other parts of plants. In 2021, we endeavored to measure the root phenotype of the soybean (*Glycine max*) and adzyki bean (*Vigina angularis*) germplasms for the first time across international genebanks [22,23]. Specifically, we cultivated 380 soybean landraces and 61 wild adzuki beans in polyvinyl chloride column (PVC) pipes until the V2 stage, when the second true leaf emerged, and we constructed images with a scanner and analyzed the root phenotypes with commercially available WinRIZO software [24]. We confirmed that eight root morphological traits (RMT) were distinctive for classification, and the accessions were divided into three clusters. Furthermore, it was feasible to provide breeders with information by selecting excellent germplasms in comparison to the *Glycine max* cv. Enrei Japanese varieties, which are known for their excellent nodulation characteristics. Additionally, these digital phenotyping techniques can be applied to various other crops conserved at the RDA-Genebank. For instance, in wheat, RGB images obtained from a digital camera can be utilized to measure agronomic traits such as tiller number per plant, canopy (including leaf number and length), phenotypic characteristics of wheat spike, and tomato internode length. In the future, RGB images captured using unmanned aerial vehicles can also be used to measure plant heights in crops such as rice, wheat, barley, and corn [25].

**Figure 6:**
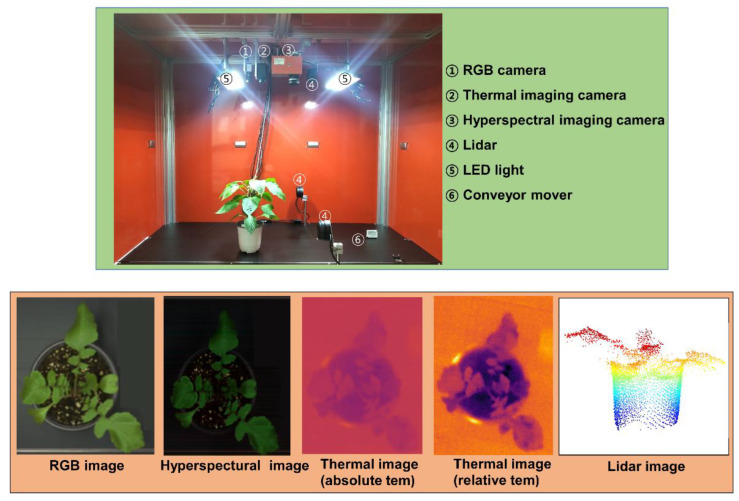
State-of-the-art types of digital equipment for researching aboveground growth of crops held by RDA-Genebank.

Hyperspectral sensors have been utilized to measure photosynthesis and respiration, which are directly related to crop growth and are currently being studied in tobacco, corn, and wheat [26]. In addition, biomass yield research has commenced by utilizing shoot color in wheat for biomass yield estimation and thermal imaging cameras for wheat tiller number [22]. Hyperspectral imaging is a nondestructive method for the early detection of crop disease and is based on the spatial and spectral information of the images. Regarding plant disease detection, hyperspectral imaging can predict disease-induced biochemical and physical changes in plants [23]. Hyperspectral imaging has the advantage of using a nondestructive method to identify diseases in the early stages of plant growth and predict physical changes in disease-infected crops [27]. While the hyperspectral method can be efficiently used to detect plant diseases, it can also be used in a high throughput setting to select resources such as disease-resistant plants, targeting a large number of resources held in genebanks. We are currently studying the growth of horticultural crops using the NDVI index of the aboveground part using a hyperspectral camera installed in a box (Figure 6). In addition, a method of measuring the height of crops using Lidar images is currently being prepared. Our final goal is to record agricultural traits in a digital way that complements the existing conventional methods and provide them to the consumer by quickly compiling a large amount of information.

**Figure 7:**
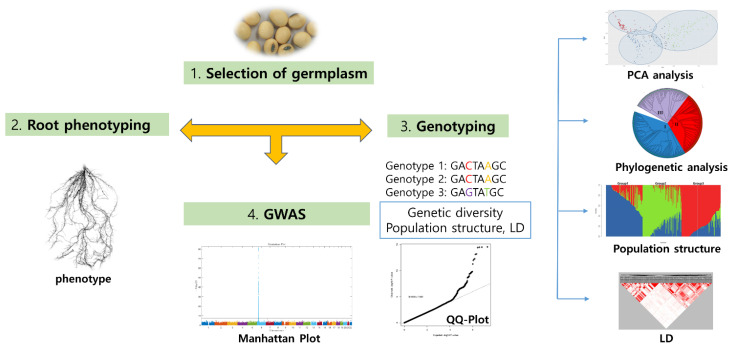
Flow chart of currently proposed germplasm research conducted by RDA-Genebank [30].

The augmentation of phenotype data with existing characterization information, as well as the integration of genomic, phenomic, and other molecular data, can significantly aid researchers in selecting optimal accessions for breeding and elucidating various evolutionary characteristics [28]. In the case of rice, the combination of phenotyping data with genome-wide association studies has proven useful in understanding the genetic variation occurring in the crop [29]. At the RDA-Genebank, we conducted a genome-wide association study (GWAS) using root phenotype data and 180K single-nucleotide polymorphisms (SNPs), leading to the identification of 112 SNP loci related to seven root traits and the selection of 55 putative candidate genes (Figure 7) [30]. Phenomics is a vital component of the recently proposed concept of ‘PANOMICS,’ which seeks to integrate multiple ‘-omics’ datasets for faster analysis and improved germplasm quality [31]. Therefore, in the near future, the RDA-Genebank plans to provide germplasm information in a combined format for end-users to facilitate the selection of appropriate germplasms.

## 6. Conclusions

Given the accelerating loss in biodiversity worldwide due to changing climatic conditions and anthropogenic activities, the importance of agricultural genetic resources is becoming more prominent. At plant genebanks throughout the world, continuous efforts are being taken towards improving the quality and quantity of germplasm collection along with ease of access. The germplasms we conserve at the National Agrobiodiversity Center are continuously investigated for agricultural traits and building an updated database. Recently, we have also concentrated on methods for investigating agricultural traits by applying digital techniques. We expect information on the germplasms conserved in the RDA-Genebank to be made accessible at an early juncture to researchers all over the world. Ensuring timely access to vital genetic resources will foster international collaboration and contribute to the advancement of research and agricultural development worldwide. By promoting transparency and facilitating the exchange of germplasms, plant biologists can collectively work towards enhancing global food security, biodiversity conservation, and sustainable agriculture. As custodians of invaluable genetic resources, the RDA is committed to promoting their widespread availability and fostering fruitful partnerships with researchers and institutions across the globe.

## Figures and Tables

**Table 1:** Classification by institution of germplasms distributed in RDA-Genebank for the past 20 years from 2001 to 2020.

RDA	University	Local Agronomy Institute	Institute	RDA-Genebank	Seed Company	Overseas	Etc.	Total
179,924	122,103	42,157	12,464	378,113	19,607	5639	10,273	770,280

## Data Availability

Not applicable.
